# Recent advances in nutrition

**Published:** 2017

**Authors:** Dominic King, David Aldulaimi

**Affiliations:** 1 *Heart of England Foundation Trust, UK*; 2 *South Warwickshire Foundation Trust, UK *

 Inflammatory bowel disease (IBD) is frequently associated with nutritional deficiencies and weight loss (1). TNFα has a key role in regulating inflammation, and levels of TNFα are increased in the mucosa of patients with IBD compared to controls. The treatment of IBD has been revolutionized by the introduction of anti-TNFα treatment. TNFα also inhibits the anabolic effects of IGF -1.

This Hungarian based single center prospective study investigated the effect of infliximab (IFX) and adalimumab (ADA) on the nutritional status of patients with IBD, when administered according to national guidelines over a 12-week period. Forty patients were recruited (33 with Crohn’s disease and 7 with ulcerative colitis). Nutritional assessment was performed by dietician-led interviews and body composition analysis. 

Treatment with IFX and ADA was associated with increased energy and protein intake, an increase in body mass index and muscle mass and reduced sarcopenia. The investigators concluded that IFX and ADA had a beneficial effect on the nutritional status of patients with active IBD.

Long-term home parenteral nutrition (HPN) is a lifesaving treatment for patients with intestinal failure. Treatment is frequently complicated by catheter related blood stream infections (CRBSI). Long-term home parenteral nutrition for pediatric patients is particularly challenging and the risks of CRBSI remain uncertain in this population.

This single center retrospective study reported the incidence of CRBSI in 8 pediatric patients treated with HPN by a pioneering service from 2006 to 2012. Eight patients, aged 1 to 8, were included. Indications for treatment included micro-villous inclusive disease and short bowel syndrome. Seventy-four catheter replacements were required (60 Hickman). The CRSBI rate was 2.9 per 1,000 days. CRSBIs were responsible for 60 hospital admissions, with a median duration of 182 days. Staphylococcus species were the most frequent organism isolated.

The authors conclude that well-organized teaching supports the safe provision of HPN (2).

Severe acute pancreatitis (SAP) is a significant cause of morbidity and mortality. Enteral nutritional therapy reduces loss of mucosal integrity compared to parenteral nutrition or fasting. Retained mucosal integrity prevents bacterial translocation and sepsis complications, including pancreatic necrosis. Enteral nutrition with nasogastric (NG) and naso-jejunal (NJ) feeding has been studied in SAP, and NG feeding is considered safe. The definition of SAP was modified in 2012. This study compared NG and NJ feeding in SAP according to the new criteria.

Studies were identified in PubMed, EMBase and Cochrane central register databases. The authors included only studies that were randomized control trials, included patients with SAP according to the 2012 criteria, compared NG and NJ feeding and reported mortality. Only 4 studies were identified with a total of 237 patients, 122 randomized to NG and 115 to NJ feeding.

On meta–analysis, the authors reported no significant difference in mortality, infectious complications or length of stay. This meta–analysis suggests that NG feeding is safe in patients with SAP according to the new 2012 criteria (3).

Irritable bowel syndrome (IBS) is a common condition that is frequently treated with dietary modification. Recent studies have suggested that a gluten free diet (GFD), in the absence of either coeliac disease (CD) or wheat allergy, can result in symptomatic improvement. These studies have typically been performed in Western populations and have suggested a non-coeliac gluten sensitivity. 

In this study, the authors report the results of a study that recruited patients, diagnosed with IBS according to the Rome III criteria, to a double blind placebo controlled trial in Mumbai. Patient were interviewed by a dietician and advised to follow GFD for four weeks. Patients who responded were then randomized to either a GFD or gluten containing diet (GCD). Symptoms including bloating and abdominal pain were recorded on a visual analogue score.

Totally, 180 patients were recruited in the study. Sixty-five responded to GFD. Thirty-one patients were then randomized to continue GFD and 34 patients remained on GCD. The patients allocated to GCD had significantly more symptoms than those randomized to GFD.

This study suggests that GFD may be beneficial for patients diagnosed with IBS from the Indian sub-continent (4).

Non-alcoholic fatty liver disease (NAFLD) is increasingly recognized as the most important cause of chronic liver disease in many developed countries. The association of NAFLD with diabetes and obesity has led researchers to investigate dietary modification as a possible treatment. Low carbohydrate diets (LCD) have been investigated in several studies. In this meta-analysis, the authors reviewed studies investigating LCD for treatment of NAFLD. The authors were unaware of any similar meta-analysis that had been published.

Studies were included if patients had been diagnosed with NAFLD. The study provided exact details about the dietary intervention, carbohydrate provided less than 50% of the dietary energy and details of at least one liver enzyme was provided. A total of 10 studies were identified for meta-analysis with 238 patients. Four studies assessed intra-hepatic lipid content (IHLC), in a total of 50 patients assessed by biopsy or imaging. Five studies were performed in the United States, four in Europe and one in Asia.

Meta-analysis results showed no significant improvement in liver function tests but did report a significant reduction in intrahepatic content. This meta-analysis suggests that LCD may have a beneficial effect on IHLC (5).
